# Torsade de Pointes due to Methadone Use in a Patient with HIV and Hepatitis C Coinfection

**DOI:** 10.4061/2010/524764

**Published:** 2010-12-30

**Authors:** Jinu John, Xixi Amley, Gabriel Bombino, Chaim Gitelis, Bernard Topi, Gerald Hollander, Joydeep Ghosh

**Affiliations:** ^1^Department of Internal Medicine, Maimonides Medical Center, 4802 Tenth Avenue, Brooklyn, NY 11219, USA; ^2^Division of Cardiology, Maimonides Medical Center, 4802 Tenth Avenue, Brooklyn, NY 11219, USA

## Abstract

We present a case of Torsade de Pointes secondary to multiple factors including patient susceptibility and iatrogenic influences. Contributing causes are presented, and the approach to treatment is discussed.

## 1. Case Report

A 50-year-old male with a past history of HIV infection, acquired immune deficiency syndrome (AIDS), pneumocystis carinii pneumonia, hepatitis C, and hepatoma (postradiofrequency ablation) presented to the emergency room for weakness and syncope which occurred in the bathroom while straining to defecate. He denied palpitation, chest pain, or shortness of breath prior to losing consciousness. There was no history of seizure disorder. The following day while walking to the hospital, he developed lightheadedness again which was associated with diaphoresis and had to hold onto a pole for support for two minutes before feeling able to continue. There was a history of cocaine and IV heroin abuse (discontinued two years ago). 

**Figure 1:**
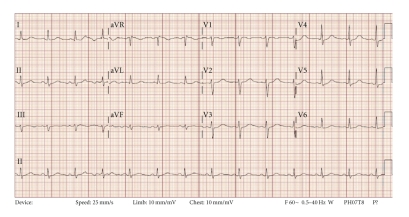
Baseline EKG was acquired about 14 months prior to the syncope episodes. QTc 455 ms.

**Figure 2:**
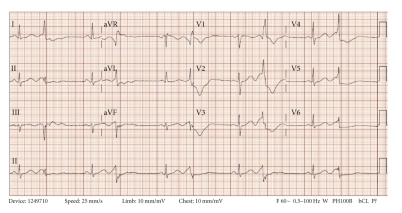
Initial 12 lead EKG in emergency room showing ventricular bigeminy. QTc was 550 ms.

**Figure 3:**
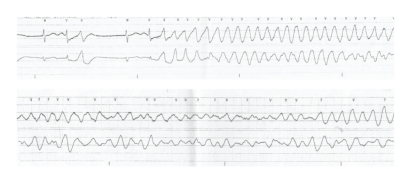
EKG tracing in emergency room showed short-long-short sequence of RR cycle followed by TdP. The patient had two episodes of syncope while he had TdP. The above two tracings are from one of the two episodes.

**Figure 4:**
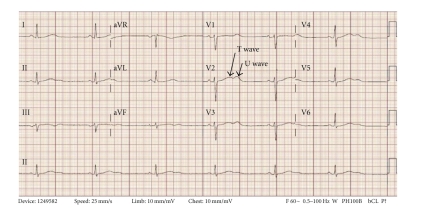
Sinus bradycardia with heart rate of 38 beats per minute occurred three days after admission. QTc 571 ms. Also noted are pathological T-U waves.

**Figure 5:**
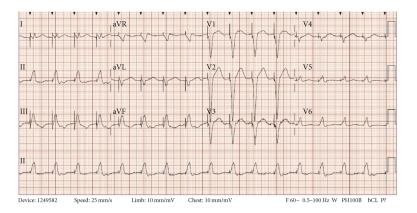
Intravenous temporary pace maker was inserted for sinus bradycardia with long QTc. To prevent TdP, pacing rate was set at 100 beats per minute.

**Figure 6:**
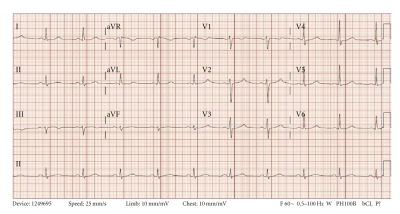
23 days after admission, the EKG showed a QTc of 492 ms prior to ICD placement. The pathological T-U waves are no longer seen.

Prehospital medications included lamivudine/zidovudine, atazanavir, trimethoprim/sulfamethoxazole, and methadone. He was on antiretroviral drugs for 10 years, and the methadone dosage was increased to 40 mg/day two months prior to this presentation. On admission in the ER, he had a regularly irregular pulse and a blood pressure of 124/60. Physical examination was unremarkable except for ecchymosis in the left periorbital region. Relevant lab reports produced the following results: MCV 131 FL, platelet count 82,000/mm^3^, AST 176 IU/L, alkaline phosphatase 140 IU/L, ALT 104 IU/L, albumin 3.2 g/dL, and INR 1.3. Serum potassium, magnesium, corrected calcium, and cardiac enzymes were within normal range. The patient's chest X-ray was unremarkable. EKG recorded 14 months prior to the ER presentation showed sinus rhythm and QTc 455 ms (Figure 1). The initial EKG on presentation showed ventricular bigeminy (Figure 2) with QTc 550 ms. In the ER, he developed two episodes of TdP (Figure 3) each resolving spontaneously, although the patient became unconscious for approximately two minutes during each episode. He was given 2 g Magnesium IV and Amiodarone IV push 150 mg twice. The patient was transferred to cardiac ICU. HIV medications were switched to raltegravir, emtricitabine/tenofovir; methadone was discontinued. Three days later, he developed sinus bradycardia at 38 beats per min (bpm) with QTc 571 ms (Figure 4). To shorten his QTc and prevent further episodes of TdP, the patient underwent a temporary pacemaker insertion and was paced at a rate of 100 bpm (Figure 5). After two days, his QTc slowly decreased and sinus bradycardia resolved. 23 days following the modification of the medications administered to the patient, the EKG still exhibited prolonged QTc (Figure 6). As the patient was considered at high risk to develop TdP, he underwent an implantable cardioverter defibrillator (ICD) placement. He is currently being followed with no further episodes of TdP.

## 2. Discussion

TdP belongs to a group of polymorphic ventricular tachycardia characterized by varying QRS amplitudes that appear to twist around an isoelectric line in the setting of long QTc as revealed in this patient's baseline EKG. TdP is commonly preceded by long-short R-R cycle which is followed by a late premature ventricular complex. Often, early after depolarization manifested as T-U waves in EKG precede TdP. In patients with prolonged QTc, the presence of pathological T-U waves (Figure 6) might be the only warning sign of imminent TdP [1].

 Very often, the cause of TdP is multifactorial. The common risk factors of TdP include (a) female gender, (b) heart disease, (c) electrolyte abnormalities, (d) bradyarrhythmias, (e) stroke, and (f) a significant universe of drugs that lead to QT prolongation [2–4]. The upper limit of the duration of QTc is often considered to be 430 ms for adult males and 450 ms for adult females, with a normal range of plus or minus 15 percent. QTc prolongation is defined as a QTc of more than 450 ms for adult males and 470 ms for adult females [5]. Studies have shown that QTc prolongation is an independent predictor of cardiovascular mortality, especially from ventricular arrhythmias such as TdP [6].

There is an increased prevalence of QTc prolongation in patients with active HIV infection. Sani and Okeahialam showed that prolonged QTc was present in 28% of such patients. In patients with AIDS, the prevalence increased to 45% [7]. In patients with HIV and autonomic neuropathy, the prevalence of QTc prolongation is reported to be 65% [8]. The exact reason for such correlation between QTc prolongation and HIV infection is still a topic of debate. But chronic HIV infection itself and many drugs used to treat HIV and other opportunistic infections are plausible explanations. In contrast, Charbit et al. suggested that QTc prolongation is related to the duration of a patient's HIV infection rather than medications used during the treatment of HIV or associated opportunistic infections [9]. Protease inhibitors (PIs) are a group of medications implicated in QTc prolongation because of the in vitro inhibition of the hERG (human ether-a-go-go-related gene) current [10]. But Charbit et al. concluded that protease inhibitors are not independently associated with QTc prolongation [9]. 

Methadone is a very frequently used drug in the management of pain and heroin addiction. Methadone causes the blockade of hERG current and causes clinically significant QTc prolongation. Martell et al. found a 10.8 ms increase in QTc within 2 months of starting methadone in a group of heroin users [11]. The prevalence of methadone-induced QTc prolongation ranges between 16% and 33%, and this can happen within a wide dose range of methadone (29–1690 mg/day) [12]. But methadone doses less than 40 mg/day do not commonly cause QTC prolongation [13]. QTc prolongation caused by methadone is dose dependant and often recent dose escalation is associated with TdP. Methadone is metabolized by CYP3A4, which is also induced by PIs. Patients, when started on PIs, might require escalation of the methadone dose for symptom control which can further prolong QTc. Methadone is also known to cause appearance of pathological U waves in EKG which may be an indicator of imminent TdP in some cases [14].

Hepatitis C is indicated as an independent risk factor for QTc prolongation. Nordin et al. investigated the impact of Hepatitis C and HIV coinfection on QTc prolongation and suggested that hepatitis C co-infection nearly doubled the risk of QTc prolongation in HIV positive patients. Liver cirrhosis, low albumin, and high AST predict QTc prolongation. These three factors tend to cluster with Hepatitis C which explains the increased risk of QTc prolongation associated with Hepatitis C infection [15]. 

The above case shows how multiple contributing factors can cumulatively cause TdP in patients who already are at risk due to genetic predisposition. This patient had a long QTc to start with and also had HIV and Hepatitis C/liver disease. He also was on antiretroviral medications for 10 years. Methadone dose escalation might have prolonged the QTc further and caused appearance of T-U waves. In this setting, a critically timed premature complex triggered off TdP. As the patient had multiple risk factors and continued to have an abnormal QTc despite changing medications, he was considered to be at high risk for further development of TdP, and the decision of utilizing an ICD was made. 

This case also highlights the need for carefully monitoring susceptible patients treated with methadone for potentially dangerous QT prolongation which can occur even after the patient has been on therapy for some time. Patients should also be made aware of relevant drug interactions and the importance of reporting episodes of dizziness or near syncope. Recent guidelines suggest getting a baseline EKG before starting patients on methadone. After initiation of therapy, EKG should be repeated to look for QTc prolongation within one month and then annually even if patients are asymptomatic. Discontinuation of methadone should be considered if QTc > 500 ms [16].
